# Patient-Derived Cellular Models for Polytarget Precision Medicine in Pantothenate Kinase-Associated Neurodegeneration

**DOI:** 10.3390/ph16101359

**Published:** 2023-09-26

**Authors:** Mónica Álvarez-Córdoba, Marta Talaverón-Rey, Suleva Povea-Cabello, Paula Cilleros-Holgado, David Gómez-Fernández, Rocío Piñero-Pérez, Diana Reche-López, Manuel Munuera-Cabeza, Alejandra Suárez-Carrillo, Ana Romero-González, Jose Manuel Romero-Domínguez, Alejandra López-Cabrera, José Ángel Armengol, José Antonio Sánchez-Alcázar

**Affiliations:** 1 Andalusian Centre for Developmental Biology (CABD-CSIC-Pablo de Olavide University), 41013 Seville, Spain; malvcor@upo.es (M.Á.-C.); mtalrey@upo.es (M.T.-R.); spovcab@upo.es (S.P.-C.); pcilhol@upo.es (P.C.-H.); dgomfer1@acu.upo.es (D.G.-F.); rpieper@alu.upo.es (R.P.-P.); dreclop@alu.upo.es (D.R.-L.); mmuncab@upo.es (M.M.-C.); asuacar1@alu.upo.es (A.S.-C.); aromgon1@upo.es (A.R.-G.); jmromdom@upo.es (J.M.R.-D.); alopcab2@alu.upo.es (A.L.-C.); 2 Department of Physiology, Anatomy and Cellular Biology, Pablo de Olavide University, 41013 Seville, Spain; jaarmbut@upo.es

**Keywords:** neurodegeneration with brain iron accumulation (NBIA), pantothenate kinase-associated neurodegeneration (PKAN), pantothenate kinase 2 (PANK2), pantothenate, pantethine, vitamin E, omega 3, α-lipoic acid, L-carnitine, thiamine, fibroblasts, induced neurons, precision medicine

## Abstract

The term neurodegeneration with brain iron accumulation (NBIA) brings together a broad set of progressive and disabling neurological genetic disorders in which iron is deposited preferentially in certain areas of the brain. Among NBIA disorders, the most frequent subtype is pantothenate kinase-associated neurodegeneration (PKAN) caused by pathologic variants in the *PANK2* gene codifying the enzyme pantothenate kinase 2 (PANK2). To date, there are no effective treatments to stop the progression of these diseases. This review discusses the utility of patient-derived cell models as a valuable tool for the identification of pharmacological or natural compounds for implementing polytarget precision medicine in PKAN. Recently, several studies have described that PKAN patient-derived fibroblasts present the main pathological features associated with the disease including intracellular iron overload. Interestingly, treatment of mutant cell cultures with various supplements such as pantothenate, pantethine, vitamin E, omega 3, α-lipoic acid L-carnitine or thiamine, improved all pathophysiological alterations in PKAN fibroblasts with residual expression of the PANK2 enzyme. The information provided by pharmacological screenings in patient-derived cellular models can help optimize therapeutic strategies in individual PKAN patients.

## 1. Introduction

NBIA represents a group of rare genetic neurodegenerative diseases that clinically manifest the presence of severe dystonia, rigidity, dysarthria, loss of ambulation, parkinsonism, choreathetotic movements, retinal degeneration or optic nerve atrophy, neuropsychiatric disorders and can lead to premature mortality [1]. The most frequent pathological findings are iron deposits in the basal ganglia and adjacent areas, and generalized axonal dilations (called spheroid bodies) in the central nervous system (CNS), representing degenerated neurons [2]. At present, more than 15 genes are associated with NBIA disorders [3]. However, the responsive genes of nearly 20% of the patients with clinical suspicion of NBIA are unknown.

Despite the intense efforts in research on these diseases and the proposals of new therapeutic approaches, there are still no effective treatments to halt the progression of neurodegeneration in NBIAs. Therefore, new therapeutic strategies are necessary.

Pathological variants in the pantothenate kinase 2 (*PANK2*) gene, which encodes for an essential enzyme involved in the coenzyme A (CoA) biosynthesis pathway, are one of the most prevalent NBIA subtypes; it represents nearly 50% of cases [4]. PKAN includes a continuous phenotypic spectrum with two major clinical forms: classic PKAN and atypical PKAN. Classic PKAN has an early onset in childhood (usually in the first decade of life) and a rapid neurodegenerative progression. On the other hand, atypical PKAN is characterized by a later onset (commonly in the second or third decade of life), and a slower course of the disease [5,6]. Despite this clinical classification, there are patients with early disease debut but insidious progression or late start with fast progression.

The *PANK* gene family comprises *PANK1a*, *PANK1b*, *PANK2*, *PANK 3* and *PKAN4* genes, but only pathological variants of *PANK2* cause PKAN. PANK1, PANK2, and PANK3 isoenzymes are active as dimeric complexes with different localizations in the cell. PANK2 is the only isoform to be expressed in mitochondria in humans and primates [7], whereas PANK1 and PANK3 are commonly localized in the cytosol and nucleus [8]. On the other hand, PANK4 is a pseudo-pantothenate kinase that lacks kinase activity; however, it shows phosphatase activity catalyzing the dephosphorylation of phosphopantothenate, 4′-phosphopantetheine and its derivatives [9,10].

The PANK2 enzyme catalyzes the key regulatory reaction in CoA biosynthesis in which pantothenate is converted into 4′-phosphopantothenate using ATP. The main mechanism for controlling PANK2 activity is through allosteric inhibition by acetyl-CoA and CoA thioesters [11]. Recently, Cavestro et al. have reviewed CoA biosynthesis and degradation pathways [12].

## 2. Etiopathogenesis of PKAN

### 2.1. CoA Deficiency in PKAN

**Figure 1:**
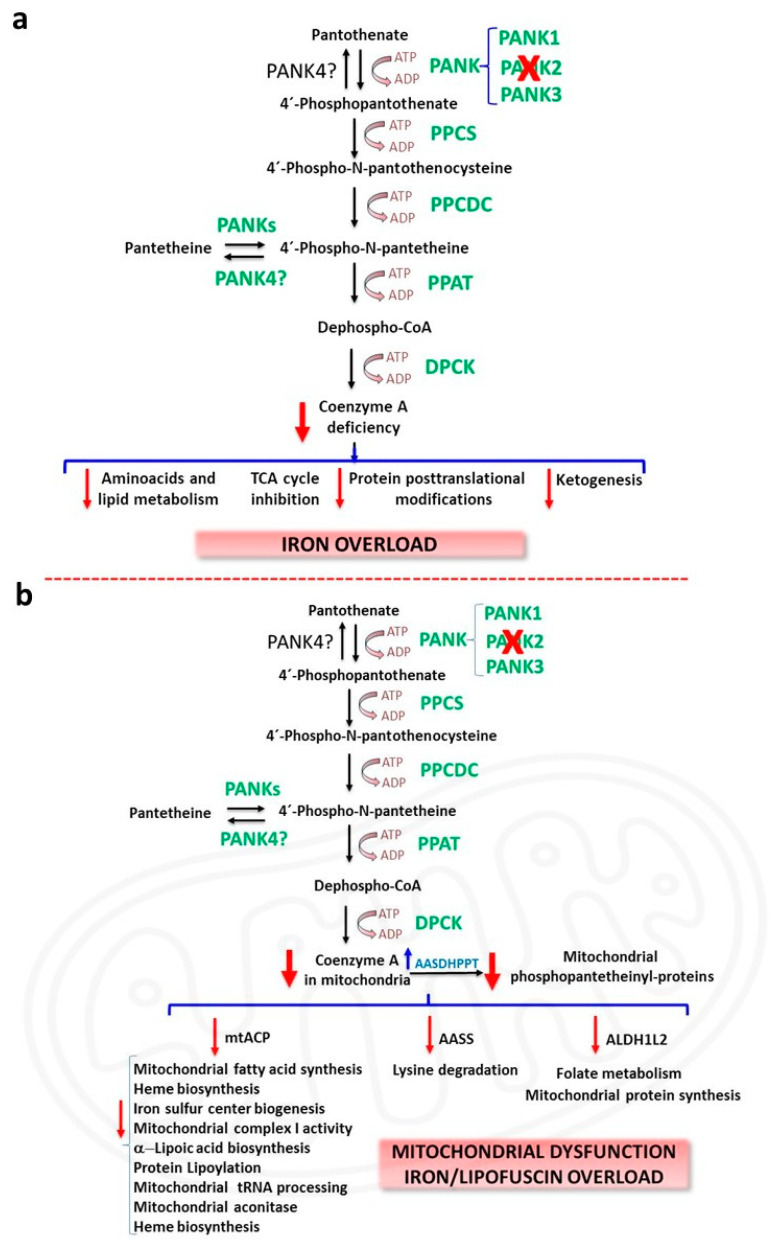
CoA deficiency in PKAN. (**a**) Etiopathogenesis of PKAN based on cellular CoA deficiency; (**b**) Etiopathogenesis of PKAN based on the deficiency of mitochondrial phosphopantetheinyl-proteins. AASDHPPT, L-aminoadipate-semialdehyde dehydrogenase-phosphopantetheinyl transferase; AASS, α-aminoadipate semialdehyde synthase; ALDH1L2, Aldehyde Dehydrogenase 1 Family Member L2 (mitochondrial 10-formyltetrahydrofolate dehydrogenase, 10-FTHFDH); DPCK, dephosphocoenzyme A kinase; mt ACP, mitochondrial acyl carrier protein; PKAN, pantothenatekinase–associated neurodegeneration; PPAT, phosphopantetheine adenylyl transferase; PPCDC, phosphopanthenoylcysteine decarboxylase; PPCS, phosphopantothenoylcysteine synthetase; TCA, tricarboxylic acid cycle.

Deficient PANK2 enzyme activity due to *PANK2* mutations is hypothesized to impair the biosynthesis of CoA, leading to multiple metabolic alterations including deficient tricarboxylic acid cycle (TCA) and cell bioenergetics, amino acids and lipid metabolism, and ketone body production [13,14] (Figure 1a). In addition, CoA also participates in protein regulation by posttranslational modifications (Acetylation, CoAlation, Acylation and 4-phosphopantetheinylation) [15]. However, the connection between CoA levels and PKAN pathomechanisms is not clear because CoA levels in PKAN patient-derived fibroblasts are similar to control cells [16,17]. Furthermore, no experimental data are available showing reduced CoA levels in PANK2-deficient human tissues. Furthermore, CoA levels were not decreased in any tissue in adult *Pank2*-KO mice [18]. These findings suggest that the increased expression levels of other PANK isoforms may compensate for the loss of PANK2 activity. Thus, expression levels of PANK1, but not PANK3, were remarkably increased in PKAN fibroblast cell lines [19]. However, a significant reduction in CoA levels was detected in mitochondrial fractions of PKAN fibroblasts [19] suggesting that a critical mitochondrial pool of CoA might be reduced in PKAN [20], and thus CoA levels in cells and tissues are unaffected as the result of the compensatory increase in the other PANK isoform activities.

The CoA compartmentalization hypothesis in PKAN is supported by the hypothesis that mitochondrial CoA supplies the 4’-phosphopantetheine cofactor for the posttranslational modification required to activate specific and essential mitochondrial proteins [20] (Figure 1b). Enzymes that catalyse sequential reactions often operate as complexes and are dependent on the covalent binding of a 4′-phosphopantetheine cofactor to specific subunits.

These 4′-phosphopantetheinyl proteins carry metabolic intermediates during sequential enzymatic reactions. The transfer of the 4′-phosphopantetheinyl cofactor from CoA is a post-translational modification [21] necessary for the transformation of apo-proteins into their full-active holo forms [21]. Thus, 4′-phosphopantetheinylation is crucial for the activity of a number of essential proteins including acyl carrier protein (ACP) which is involved in type I fatty acid synthesis (FAS) and mitochondrial ACP (mtACP) participating in type II mitochondrial FAS, α-aminoadipate semialdehyde synthase (AASS) which is implicated in lysine metabolism and 10-formyltetrahydrofolate dehydrogenase (10-FTHFDH) that presents two isoforms: cytosolic 10-FTHFDH or ALDH1L1 (Aldehyde Dehydrogenase 1 Family Member L1) and mitochondrial 10-FTHFDH or ALDH1L2 (Aldehyde Dehydrogenase 1 Family Member L2) participating in folate metabolism [21] (Figure 1b). Interestingly, mammal’s genome only encodes one unique phosphopantetheinyl transferase (PPTase), termed L-aminoadipate-semialdehyde dehydrogenase-phosphopantetheinyl transferase (AASDHPPT) [22]. This enzyme hydrolyses coenzyme A to 4′-phosphopantetheine and 3′,5′-adenosine diphosphate, and transfers the 4′-phosphopantetheinyl cofactor to a serine amino acid at the catalytic center of the apo-proteins. Crystallization studies on human PPTase have allowed a better knowledge of its substrate binding and catalytic process [23].

The fatty acid synthase (FAS) is a cytosolic multi-enzyme protein that catalyzes fatty acid synthesis from acetyl-CoA and malonyl-CoA to the corresponding acyl carrier protein (ACP) derivatives [24]. FAS consists of seven catalytic domains: acyl carrier protein (ACP), malonyl/acetyltransferase, ketoacyl synthase, ketoacyl reductase, dehydrase, enoyl reductase, and thioesterase [24,25]. As an acyl carrier, ACP depends on its phosphopantetheine cofactor which acts as a long sidearm allowing the translocation of the growing fatty acyl chain intermediate from one catalytic site to another in the FAS complex [24,25]. Cytosolic ACP forming part of the FAS complex has been described as the main acyl-carrier protein participating in fatty acid biosynthesis. However, it has been identified a mtACP protein different from cytosolic ACP, which also carries a 4′-phosphopantetheine prosthetic group [26,27]. The presence of an independent pathway for mitochondrial fatty acid synthesis suggests that it may be essential for the specific mitochondrial phospholipid metabolism [28,29]. Furthermore, type II mitochondrial FAS is the sole source of the octanoic acid precursor required to produce the lipoic acid cofactor essential for several mitochondrial proteins [30]. The localization of PANK2 in mitochondria and the modulation of PANK2 activity by acyl-CoA derivatives may also have biological significance for the development of a separate fatty acid biosynthesis pathway in type II mitochondrial FAS [31].

In agreement with the CoA compartmentalization hypothesis, decreased PANK2 expression levels and reduced mitochondrial CoA levels [19], were associated with the down regulation of mitochondrial 4′-phosphopantetheinyl proteins such as mtACP, mitochondrial10-FTHFDH (ALDH1L2) and AASS in mutant PANK2 fibroblasts [32]. Interestingly, the expression levels of AASDHPPT, the enzyme that transfers 4′-phosphopantetheine from CoA to specific proteins were up-regulated, likely as a compensatory mechanism to cope with low CoA levels in mitochondria and the consequent 4′-phosphopantetheinylation defect. Furthermore, low levels of phosphopantetheinyl-proteins in PKAN fibroblasts were limited to the mitochondrial compartment since cytosolic 4′-phosphopantetheinyl proteins such as FAS and cytosolic 10-FTHFDH (ALDH1L1) showed normal expression levels [32].

Consistent with mtACP deficiency which also hinders lipoic acid biosynthesis by type II mitochondrial FAS [30], lipoylated protein levels are also predicted to be downregulated. Thus, it has been reported that pyruvate dehydrogenase (PDH) lipoylation and activity were markedly decreased in PKAN patients-derived cells [32]. Similarly, in the CoA-deficient Drosophila model, decreased mtACP levels were accompanied by reduced mitochondrial protein lipoylation and PDH activity [20]. Likewise, the activity of other lipoylated enzyme complexes such as α-ketoglutarate dehydrogenase (αKGDH) [33] are predicted to be affected since expression levels of lipoylated αKGDH were notably down-regulated in PKAN fibroblasts [32]. Lipoic acid deficiency is also predicted to affect the lipoylation and activity of three additional enzymes from the amino acid metabolism: branched-chain ketoacid dehydrogenase, 2-oxoadipate dehydrogenase, and the glycine cleavage system (GCS) [34]. However, to date there are no studies on the activity of these enzymes in PKAN.

In addition, deficiency of mt-ACP may also alter several mitochondrial processes because mtACP is implicated in essential mitochondrial functions. Indeed, mtACP, also known as NDUFAB1 (NADH:ubiquinone oxidoreductase (NDU)-FAB1), forms part of mitochondrial respiratory complex I and is required for its assembly [35,36]. Furthermore, mtACP participates in the iron-sulfur cluster (ISC) biosynthetic pathway and stability, indicating that the 4′-phosphopantetheinyl modification of mitochondrial proteins is crucial for mitochondrial iron metabolism [37,38,39]. Thus, the hypothesis proposes that mtACP deficiency leads to reduced complex I activity and ISC formation. This prediction is consistent with the observations of Jeong et al. in a PKAN mouse model [38] and Lambrechts et al. in Drosophila models of CoA deficiency [20]. Furthermore, the loss of mtACP in *Saccharomyces cerevisiae* leads to reduced ISC formation, accompanied by the inactivation of Fe-S cluster-dependent enzymes such as aconitases (which contain a Fe-S cluster cofactor), and activation of iron-responsive factors Aft1 and Aft2 [39]. Interestingly, decreased Fe-S cluster levels lead to mitochondrial iron overload [40]. In agreement with these findings, abnormal iron metabolism and downregulation of aconitase activity have been reported in patient-derived fibroblasts as well as iPSC-derived neurons [41,42].

All these predictions and observations have been also confirmed by other researchers in PKAN patients-derived cellular models [32]. Thus, mitochondrial complex I activity, expression levels of proteins involved in ISC formation as well as mitochondrial and cytosolic aconitase activities were notably decreased in mutant PANK2 fibroblasts [32].

### 2.2. Iron/Lipofuscin Accumulation in PKAN

Iron is an essential element for cell homeostasis due to its role as a versatile cofactor in many iron-containing proteins involved in cell metabolism and signalling homeostasis [43]. However, redox-active iron can participate in reactions that generate damaging ROS and consequently may promote oxidative stress, lipid/protein oxidation, nucleic acid damage [44,45], and finally cell death by ferroptosis [46]. Iron detection of PKAN brain tissues by Prussian blue staining showed extensive deposition of iron in the globus pallidus, substantia nigra and other brain areas [47].

Iron overload in PKAN has been explained by several hypotheses. One explanation is that iron overload is caused by the process of neuronal apoptosis [48]. Thus, it has been shown to increase iron uptake in ceramide-induced apoptosis [49]. However, there are no further experimental data to support this assumption. Another hypothesis connects iron metabolism dysregulation to cysteine accumulation caused by a deficient PANK2 activity [50]. After pantothenic acid phosphorylation, cysteine is conjugated to 4′-phosphopantothenate forming 4′-phosphopantothenoylcysteine, a reaction catalysed by phosphopantothenoylcysteine synthetase (PPCS). Thus, PANK2 activity deficiency could lead to the accumulation of L-cysteine and L-cysteine derivatives such as N-pantothenoylcysteine. Excess L-cysteine levels result in iron deposits due to its iron-chelating activity. Moreover, L-cysteine oxidation by iron could generate ROS resulting in increased oxidative stress [13]. In addition, L-cysteine accumulation may enhance iron-dependent lipid peroxidation, a possible secondary pathological mechanism in PKAN, leading to cell membrane damage and cell death [13]. Therefore, the toxic effects of cysteine accumulation could be a contributing factor to iron homeostasis dysregulation, increased oxidative stress and neurodegeneration in PKAN.

In dopaminergic neurons, the combination of dopamine, iron overload and high levels of cysteine can be very damaging in PKAN disease. Dopamine is a very reactive molecule, which remains stable in the acidic environment of synaptic vesicles. Nevertheless, free dopamine in the cytosol may undergo auto-oxidation reactions, generating ROS such as OH^.^, O^.−2^ and H_2_O_2_ and neurotoxic quinones [51]. The generation of neurotoxic intermediates by the interplay between dopamine and iron has been extensively examined elsewhere [52]. In brief, the two main mechanisms involved in iron-dependent dopamine neurotoxicity are the production of o-quinones by a non-enzymatic mechanism [53,54], and forming part of an intermediary iron-dopamine complex [55]. In addition, dopamine oxidation derivatives may react with L-cysteine and be converted to dihydrobenzothiazines (DHBTs) which are potent mitochondrial complex I inhibitors [56] and provoke a sustained increase in oxidative stress and apoptosis [57,58].

An alternative hypothesis states that iron can be accumulated in lipofuscin granules which are markedly increased in PKAN cells [19]. Lipofuscin (the age pigment) is a brown-yellow, electron-dense, autofluorescent aggregate that accumulates progressively in senescent cells including cardiomyocytes, hepatocytes and neurons [59]. Lipofuscin is a heterogenous mixture of oxidized proteins and lipids, metal cations, and sugar residues [59]. Approximately 2% of lipofuscin components are metals, including Fe, Cu, Zn, Al, Mn, and Ca [60]. Lipofuscin granules cannot be degraded in lysosomes or the proteasomal system which is a protease complex that recognises and degrades damaged proteins [61].

**Figure 2:**
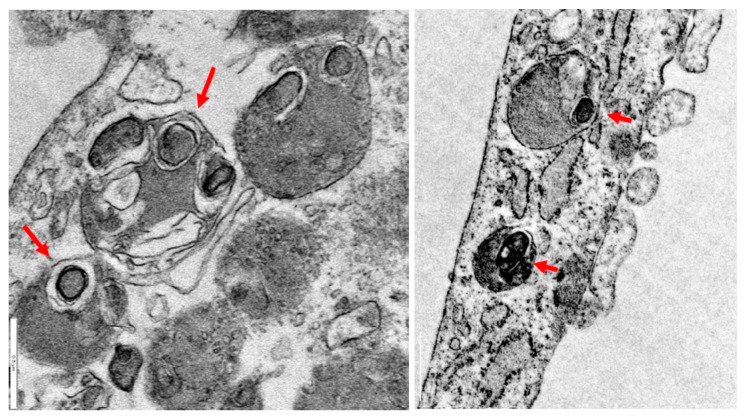
Lipofuscin formation in mitochondria in PKAN fibroblasts. Electron microscopy images of PKAN fibroblasts (unpublished data from our laboratory). Lipofuscin formation in degenerated mitochondria, red arrows. Scale Bar = 0.5 μm.

One explanation predicts that mitochondria participate in the formation of lipofuscin [62]. Supporting this hypothesis, it has been demonstrated that isolated mitochondria can degenerate to lipofuscin granules without any additional factors such as oxygen saturation or prooxidants [63]. Electron microscopy image analysis of PKAN fibroblasts showed that lipofuscin granule formation presumably takes place in degenerated mitochondria (Figure 2).

**Figure 3:**
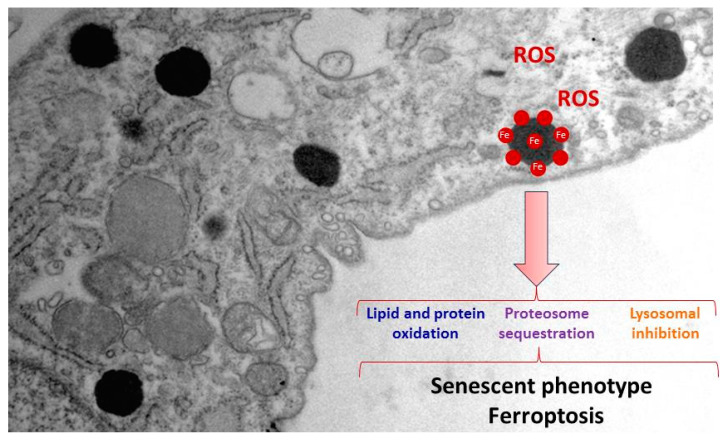
Pathological alterations of lipofuscin accumulation in PKAN cells. Increased ROS production, cellular compounds oxidation, proteasomal sequestration and lysosomal inhibition by lipofuscin granules lead to a senescent phenotype, and eventually to cell death by ferroptosis.

Lipofuscin overload is one of the best-recognised biomarkers of aging [64] and it has been demonstrated that its accumulation in PKAN cells is associated with the typical senescent morphology [19]. Several works have demonstrated that lipofuscin plays an active role in the physiopathological changes of senescent cells (Figure 3) [65,66]. Thus, it has been demonstrated that lipofuscin inhibits the proteasome [67], the main cellular protease complex for degrading damaged proteins tagged by polyubiquitin chains. Proteasomal inhibition is explained by its binding to exposed hydrophobic amino acid residues on the lipofuscin surface [68]. Moreover, lipofuscin is also able to reduce lysosomal activity by increasing lysosomal permeabilization [69,70]. Interestingly, both proteasome and lysosome inhibition strongly facilitate lipofuscinogenesis [61].

One critical factor of lipofuscin granules' cytotoxicity is due to the recruitment of transition metals such as iron [60]. Lipofuscin-trapping iron results in a redox-active surface on the granules which can catalyse the Fenton reaction (Figure 3). This quality of lipofuscin granules may increase ROS formation and oxidation of lipids and other cellular components, and eventually lead to cell death [71]. Increased oxidative stress that was further enhanced by the addition of iron has been previously reported in PKAN fibroblasts [72]. Consistent with these observations, increased levels of carbonylated proteins and mitochondrial lipid peroxidation in PKAN fibroblasts have been demonstrated [19]. Lipofuscin granules in NBIA disorders have been previously reported in conjunctival fibroblasts, retinal vessel pericytes, and macrophages [73].

Iron metabolism dysregulation in PKAN fibroblasts has been attributed to alterations of mitochondrial ISC and heme biosynthesis pathways [19,41,42,72]. ISCs are prosthetic groups bound to many subunits of mitochondrial respiratory complexes as well as cytosolic and mitochondrial aconitases [74]. For this reason, deficiency of proteins involved in ISC biogenesis may affect many mitochondrial proteins and lead to severe mitochondrial dysfunction. Another effect of this deficiency is the altered management of iron that may eventually provoke mitochondrial iron overload. In turn, high iron levels in the oxidative environment of mitochondria may trigger increased ROS production that expands and aggravates the damage [75]. In addition, ineffective mitochondrial iron utilization associated with low cytosolic free iron (cytosolic labile iron pool, CLIP) may account for the increased iron transport into the cells which progressively leads to iron overload [41]. Thus, *PANK2* silencing by siRNA in several human cell lines leads to a reduced proliferation rate accompanied by a paradoxical iron deficiency and increased Transferrin receptor protein 1 (TfR1) expression levels [76]. Considering these observations, it has been proposed the hypothesis that dysregulation of iron metabolism in mitochondria induces mitochondrial iron overload and cytosolic iron deficiency. The result is a vicious cycle characterized by increased iron uptake due to increased expression of Fe^2+^ transporters and subsequent accumulation in mitochondria and, finally, in lipofuscin granules [19,77]. This paradoxical free iron deficiency in PKAN cells may be also an important factor when considering the implementation of chelating therapies in PKAN patients.

Overall, understanding the pathomechanisms of iron overload in PKAN cells is important both for determining the etiology of PKAN and for its implications in other neurodegenerative diseases such as Parkinson’s disease (PD) and Alzheimer’s disease (AD). Thus, further studies in PKAN disease models could help to identify specific mechanisms that lead to iron metabolism dysregulation.

## 3. PKAN Disease Modeling

### 3.1. Modeling PKAN Disease in Biological Models

Although many attempts have been made to model PKAN disease in several organisms [38,78,79,80,81,82,83,84,85,86,87], they have not faithfully reproduced the main phenotypic alterations found in the disease such as brain iron overload and movement disorder symptoms, possibly because PANK2 localization in the intermembrane space of mitochondria has only been demonstrated in primates and humans. Thus, the mouse PANK2 homolog protein has been detected in the cytosol [7]. However, other researchers have described a mitochondrial localization although a mitochondrial targeting sequence has not been identified in the mouse PANK2 enzyme [88,89]; In a *PANK2* knockout mouse model, researchers found decreased weigh, retinal degeneration and azoospermia, but no movement disorders or signs of iron accumulation in the brain [82]. Nevertheless, a deficient diet in pantothenic acid provoked movement alterations in the knockout mice but iron deposition in basal ganglia was not detected [90]. For a detailed updated of PKAN murine models see [91].

Several works have been performed with the aim of generating PKAN models in Drosophila with varying degrees of success. Drosophila, has only a single PANK isoform (fumble, *fbl*) [92] and its suppression caused developmental abnormalities of the CNS [78] that were rescued by pantethine treatment [83]. However, iron deposits were not detected in neurons.

In the Zebrafish model, *PANK2* silencing by morpholinos caused malformations of the CNS, particularly in the telencephalon, and vascular structures [87]. In another study, overexpression of mutant human PANK2 and mutant zebrafish PANK2 mRNA in zebrafish embryos caused vascular and neurological defects and reduced locomotor activity [81]. Although neurological defects were expected, vascular defects had not been reported in any other model of PKAN. This finding might suggest an unknown role of PANK2 activity in vascular development.

Modeling PKAN in *S. cerevisiae* could be particularly of interest since the CoA biosynthesis pathway is highly conserved between yeast and humans. Another advantage is that yeast cultures are easy to handle and allow genetic and cellular assays to examine the consequences of CoA deficiency and evaluate therapeutic strategies. The yeast *PANK* homolog, Cab1, codifies a unique PANK enzyme which is essential for cell viability [80,93]. A recent study showed that the Cab1G315S mutant reproduces the cellular defects found in cells isolated from PKAN patients [94]. Furthermore, iron content assays revealed increased levels of intracellular iron associated with decreased expression levels of key iron uptake genes [94]. Studying the mechanisms that cause this iron dysregulation in yeasts could be of interest to understanding the role of iron overload in PKAN. Moreover, yeast models of PKAN also showed mitochondrial dysfunction characterized by low oxygen consumption rate as well as cytochrome c oxidase and NADH cytochrome c reductase activities [94]. Similarly, mitochondrial dysfunction has been proposed to be involved in PKAN etiopathogenesis in patient-derived fibroblasts [20]. For all these reasons, studies in yeast might be of help to better understand PKAN disease.

### 3.2. Patient-Derived Cellular Models

The absence of suitable animal models for the investigation of PKAN has led to the development of patient-derived cell models that can serve as an alternative and complementary approach for the investigation of the molecular mechanisms of the disease and the evaluation of possible therapies. The argument for the use of patient-derived dermal fibroblast cultures is that they can be easily obtained from skin biopsies and can be amplified using standardized cell culture protocols and shared with other investigators for further studies. In addition, numerous patient-derived fibroblast cell lines are available from various cell banks.

All these features and properties make it possible to perform a wide variety of experiments using patient-derived fibroblast cell lines. Cellular and biochemical studies of patient-derived dermal fibroblasts have provided much useful information on the pathogenic mechanisms of genetic neurodegenerative diseases [95]. The rationale for this approach assumes that, although these disorders primarily affect the CNS, cultured fibroblasts harbor the specific pathological variant (even after multiple subcultures) and can mimic the pathological alterations found in the CNS. Patient-derived fibroblast models allow controlled studies of individual strain variations and may provide essential information for understanding disease pathomechanisms and for evaluating potential therapies. Thus, cell models allow us to identify what compound and at what concentration the phenotypic alterations are corrected. In addition, this strategy considers the specific characteristics of each mutation and allows the implementation of personalized medicine strategies. However, fibroblasts are not the most appropriated model for investigating neuronal dysfunction given the morphological and functional features of these cells. Accordingly, new methodological tools have been developed for the generation of neuronal models from patients with genetic neurodegenerative disorders.

### 3.3. Induced Neurons

The generation of Induced Pluripotent Stem Cells (iPSCs) in 2006 [96] has led to numerous possibilities in the field of disease modeling, drug screening and regenerative medicine. The generation of iPSC from somatic cells of patients with neurological genetic diseases and its neuronal differentiation allows disease modeling and the examination of the underlying molecular mechanisms in the most affected cells in these disorders [97]. In this respect, iPSC generation and neuronal differentiation give the possibility to establish in vitro models of NBIA disorders including PKAN. Apart from two-dimensional iPSC cultures, it is possible to obtain three-dimensional organized tissues, known as organoids. This model recapitulates features of human organs (cellular organization and architectures) [98]. Human iPSC reprogramming, combined with 3D brain organoid techniques, may serve as a preclinical stage to reduce the translational delay between animal model studies and human clinical trials. However, iPSC generation has several drawbacks such as the protocols being time-consuming, expensive and complex [99]. Furthermore, genetic instability, the risk of generating tumors and mitochondrial DNA alterations are additional obstacles on iPSC development [100].

Recently, the combination of lineage-specific transcription factors has made it possible to transdifferentiate somatic cells directly into another. For example, dermal fibroblasts can be converted into neuronal cells without bypassing an induced pluripotent state [101]. Direct transdifferentation of murine embryonic and postnatal fibroblasts into induced neurons (iNs) was first performed by Wernig and colleagues in 2010 by combining three proneural factors (Ascl1, Brn2 and Myt1l) [102]. Later, the addition of the basic helix-loop-helix transcription factor NeuroD1 allowed the conversion of fetal and postnatal human fibroblasts into iNs [103].

After these pioneer works, new tools and approaches have been developed with the aim of improving the efficiency of neuronal conversion. For instance, the addition of micro RNAs (miRNAs) such as miR-9/9* or miR-124 to the proneural genes combination resulted in the transdifferentation of human fibroblasts to functional neurons [104]. Later, it was shown that conversion efficiency, one of the main challenges of direct reprogramming, increased significantly by the combination of small molecules, proneural growth factors, and the silencing of barriers that inhibit reprogramming such as the RE-1 silencing transcription factor (REST) complex [105,106,107].

Direct reprogramming has several benefits with respect to the generation of iPSCs-derived neurons (indirect reprogramming), such as the short-time requirements and the relative simplicity of the protocols [101]. Furthermore, iNs unlike iPSCs, maintain the ageing [108] and epigenetic marks of the donor [109,110], making them attractive models for the investigation of neuronal pathophysiology in age-associated disorders. Moreover, it has been demonstrated that iNs obtained through in vivo direct reprogramming, unlike human iPSCs, do not form tumours [111], suggesting that they could be suitable for cellular regenerative therapies [112]. Thus, iNs obtained by direct reprogramming can be used in cell replacement therapy, both by in vivo reprogramming or transplantation following direct conversion in vitro. The conversion of local non-neuronal cells towards a neuronal phenotype is a promising approach for neurodegenerative disease treatment. Thus, it has been shown that endogenous mouse astrocytes can be directly converted into neurons in situ [112]. In a PD mouse model, direct conversion of dopaminergic neurons from striatal astrocytes has been performed in vivo [113]. Although this therapeutic strategy is in its initial stages, it represents the most promising approach to translating neuronal reprogramming to clinical interventions [114].

Direct reprogramming of human adult fibroblasts into iNs has been used to study several neurodegenerative diseases such as NBIA disorders [19,32,42,115,116,117], Parkinson's disease (PD) [118], Huntington disease (HD) [119], myoclonic epilepsy with ragged red fibers (MERRF) syndrome [120], as well as mitochondrial encephalomyopathy, lactic acidosis and stroke-like episodes (MELAS) syndrome [121]. However, iNs generated using direct transdifferentation have also several disadvantages. For instance, maintaining iNs in culture is difficult, since cell death can be observed from 30 DPI (days post-infection). This limitation may hamper electrophysiological characterization of iNs since action potentials have been only detected at 80–100 DPI [105]. Furthermore, cultured iNs form clusters during the transdifferentation process making difficult the isolation of single cells for specific assays.

In summary, the generation of iNs by indirect or direct reprogramming from patient-derived fibroblasts represents a very useful tool for both understanding the pathogenesis of these disorders and finding new therapeutic approaches. Interestingly, reprogramming of fibroblasts into dopamine or GABAergic o neurons will provide more information about the complex pathological connections among neurotransmitters, iron and other metabolic intermediates.

### 3.4. Alterations in Cellular Models of PKAN

Dysregulation of iron metabolism and increased oxidative stress in PKAN-patients-derived fibroblasts were previously reported [72]. Furthermore, iNs differentiated from PKAN-derived iPSC displayed mitochondrial alterations with aberrant cristae morphology and reduced mitochondrial membrane potential [41]. Interestingly, patient-derived neurons also manifest mitochondrial bioenergetics deficiency and altered electrophysiological patterns, along with dysregulation of cytosolic iron homeostasis, mitochondrial iron-dependent pathways and increased oxidative stress. Furthermore, iPSC-derived astrocytes and neurons derived from PKAN patients also showed iron overload, thus mimicking the human pathological phenotype [42,115,116].

Supporting these findings and the suitability of cell models, it has recently been described that patient-derived fibroblasts harboring several *PANK2* mutations display many of the pathological features of the disease such as intracellular iron/lipofuscin accumulation, increased oxidative stress and mitochondrial dysfunction [19,32,122,123].

## 4. Therapeutic Strategies for PKAN

At present no efficient therapy is available for PKAN. Thus, current treatments are aimed at controlling patient symptoms [1]. Although clinical trials with several compounds are in progress, PKAN treatments primarily aim to control the main disease symptoms: spasticity, seizures, dystonia, or psychiatric disorders [124]. Nevertheless, several promising therapeutic approaches are currently in progress [12,91]. These treatments can be summarized in four categories: (1) iron chelation to eliminate iron accumulation in the brain; (2) metabolite supplementation to correct metabolic deficits in the CoA pathway; (3) PANK isoforms activation to restore CoA biosynthesis; and (4) gene therapy by introducing the wild-type *PANK2* gene. However, some of these therapies have not been successful, whereas others are under evaluation. For a detailed updated of current PKAN treatment approaches see [12,91,124,125].

It is noteworthy that despite the importance of autophagy in neuronal homeostasis and pathological processes such as neurodegeneration [126], there are few studies addressing autophagy modulation in PKAN disease models. Recently, Huang et al. have shown that fumble (fbl), the human *PANK2* homolog in Drosophila, interacts genetically with PINK1 (PTEN-induced putative protein kinase 1), a key protein involved in the selective autophagy of mitochondria (mitophagy) [127]. In addition, mitochondrial fumble overexpression rescued PINK1 loss-of-function defects such as mitochondrial dysfunction. Interestingly, vitamin B5 derivatives restored CoA/acetyl-CoA levels and mitochondrial function, reversing the PINK1 deficiency phenotype [127].

### 4.1. Strategy for Finding Alternative Treatments for PKAN Using Patient-Derived Cellular Models

A key finding to support the utility of cellular models in PKAN research was that the supplementation with pantothenate, the substrate for the PANK2 enzyme, was able to increase PANK2 expression levels in patient-derived fibroblasts carrying pathologic variants with residual enzyme levels [19]. Moreover, the pantothenate-mediated up regulation of PANK2 levels was accompanied by the correction of all pathological alterations associated with PKAN such as iron/lipofuscin overload, increased lipid peroxidation and impaired mitochondrial bioenergetics. Furthermore, the positive effect of pantothenate was confirmed in iNs generated by direct reprogramming of PKAN fibroblasts [19]. These observations suggest that cell models may be a useful tool to identify patients with PANK2 mutations that respond in vitro to pantothenate supplementation. More importantly, these observations support the possibility of their treatment with high doses of pantothenate. In addition, these results suggest that personalized screening strategies in PKAN may facilitate the detection of more pharmacological chaperones (PCs) capable of increasing and stabilizing the expression levels and activity of the mutant PANK2 enzyme in specific mutations.

Many mutations in human diseases provoke the destabilization of the mutant proteins. Curiously, compounds that work as PC can rescue the activity of unstable proteins [128,129,130]. However, individual patients will be only suitable for therapy with PC depending on their specific genotype [131]. Supporting this assumption, it has been shown that several PANK2 pathological variants, but not all, can be rescued by pantothenate [19]. Therefore, a strategy for selecting more positive PCs in PKAN cellular models can lead to the identification of potential therapeutic alternatives in patients harboring specific mutations. Following this approach, several rare diseases can be already treated with PCs [132]: For Gaucher disease, Diltiazem, an antihypertensive drug [133]; for cystic fibrosis, Doxorubicin, an anti-cancer anthracycline, for cystic fibrosis [134]; for Pompe disease, Acetylcysteine, a mucolytic agent [135]; for Fabry and Gaucher disease, Ambroxol, another mucolytic agent [136]; for hyperinsulinemic hypoglycemia, Carbamazepine and dibenzazepine, [137]; for GM2 gangliosidosis, Pyrimethamine, an anti-parasitic drug [138]; and for Pendred syndrome, Salicylate, a well-known anti-inflammatory agent [139]. For PKAN disease, an allosteric brain-permeable PANK activator (PZ-2891) has been found [84]. Interestingly, a knockout mouse model of brain CoA deficiency under PZ-2891 therapy showed weight gain, improved locomotor activity and extended life span [84]. The aim of this therapeutic approach is to compensate for the loss of PANK2 by the activating of the other PANK isoforms [84].

### 4.2. Precision Medicine in PKAN

Precision medicine is an emerging approach that considers the adaptation of clinical management to the genetic characteristics of each patient. Clinical precision medicine for the management of genetic neurodegenerative disorders seems a more rational strategy in contrast to the traditional “one drug fits all patients” approach [140]. In fact, genetic neurodegenerative diseases can present heterogeneous clinical characteristics even in patients carrying the same disease or pathological variant. Furthermore, as several metabolic or signaling pathways can be secondarily affected it is highly unlikely that patients can benefit from a single drug. Genetic neurological diseases are promising models for precision medicine due to the increasing knowledge of the genetic basis of the disease and clinical classification, the increased number of biomarkers, and the existence of possible disease-modifying treatments [141].

In this context, precision medicine strategies using patient-derived fibroblasts and iNs could help optimize therapeutic approaches in PKAN.

Strategies based on precision medicine are currently applied in different health disciplines such as cardiology, nutrition, and oncology, as well as in rare diseases [142,143]. In neurodegenerative diseases, the first approaches based on precision medicine have been more relevant in Alzheimer’s disease (AD). Thus, anti-amyloid-β monoclonal antibody therapy is now being tested in patients with mutations known to cause AD with the aim of preventing neurodegeneration in patients with similar genetic alterations (ClinicalTrials.gov number NCT01760005, accessed on 5 May 2023). In addition, *APOE* (apolipoprotein E) variants can identify individuals at higher risk for AD [144], making them interesting biomarkers for earlier diagnosis, and the implementation of treatment and/or prevention strategies. Today, Parkinson's Disease (PD) is treated as one clinical entity, but many researchers emphasise that PD encompasses different sub-groups that can benefit from the approaches of precision medicine [145]. However, the complex nature of PD and AD, together with clinical phenotypic heterogeneity, present significant challenges to successfully implementing personalized medicine in these diseases.

**Figure 4:**
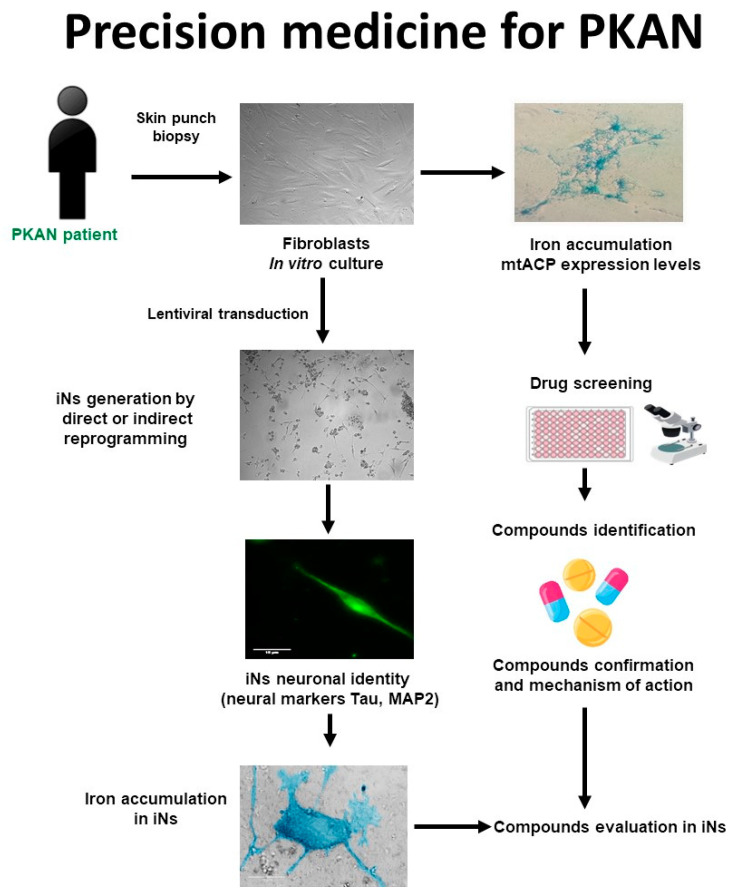
Cell-based disease modeling and drug screening approach in PKAN. PKAN patient-derived cellular models, fibroblasts, and iNs, can be useful tools for mimicking pathophysiological alterations of the disease and screening potential therapies. iNs = induced neurons; mtACP= mitochondrial acyl carrier protein.

The main phases of a personalized medicine approach applied to PKAN are illustrated in Figure 4. First, a skin biopsy is performed to generate fibroblast cultures. Subsequently, fibroblasts are characterized by examining the main alterations of PKAN disease such as iron/lipofuscin accumulation, lipid peroxidation, senescent morphology, and mutant protein expression levels. In addition to verifying PANK2 function, the expression levels of downstream proteins such as mtACP are also evaluated. Next, pharmacological screening is carried out to identify the compounds capable of correcting the alterations detected. In parallel, induced neurons are generated by indirect or direct reprogramming, verifying that they express the neuronal markers. Finally, the positive compounds identified in the fibroblast screening are evaluated in the induced neurons.

Using this strategy, 7 positive commercial supplements (pantothenate, pantethine, vitamin E, omega 3, α-Lipoic acid, L-carnitine, and thiamine) have been recently identified [122,123]. All of them were able to eliminate iron/lipofuscin accumulation, increase PANK2 and mtACP protein levels, and correct the altered phenotype in responsive mutant cells.

The rationale of pantothenate supplementation assumes that mutant enzymes may function better with higher substrate concentrations. The ability of high-dose pantothenate supplementation to improve the activity of a functionally deficient PANK enzyme is supported by in vitro studies where the affinity of the enzyme for pantothenate can be low but the reaction is still functional [146]. These observations are interesting because they indicate that pantothenate supplementation at high doses may be clinically useful for patients carrying pathological variants with residual PANK2 expression levels and/or activity. However, this therapeutic strategy is not effective in patients carrying frameshift mutations causing termination codons in both alleles that encode the expression of an incomplete/truncated protein. For this reason, in vitro evaluation of the effect of pantothenate supplementation on patient-derived cells may provide valuable information on the response of specific pathological variant subgroups. Furthermore, it is necessary to check whether pantothenate treatment can reach the proper concentration to achieve the desired functional effects in the human brain in vivo. A strategy to solve this difficulty would be to perform combined treatments with pantothenate and other pantothenate derivatives such as pantethine with the aim of increasing pantothenate concentrations in the blood and in the brain.

Pantethine is a physiological compound synthesized from pantothenic acid and cysteamine, participating as a metabolic intermediate in the biosynthesis of CoA. Pantethine treatment can increase pantothenate levels in the blood because it is highly unstable, and it is rapidly transformed into pantothenate and cysteamine [147,148]. Pantethine supplementation has been shown to rescue PKAN phenotypes in several biological models such as bacteria [149], Drosophila [83], zebrafish [87] and mice [79]. The therapeutic potentiality of pantethine in PKAN has been mainly evaluated in animal models, although the compound has been used as a lipid-lowering agent in clinical studies [150]. Recently, the safety and efficacy of pantethine (60 mg/day during 6 months) in fifteen children with PKAN have been evaluated [151]. The conclusions of this study were that pantethine supplementation did not alter serum CoA levels or improve clinical symptoms. The poor therapeutic efficacy of pantethine in PKAN patients in this study may be due to (1) the low number of patients under treatment; (2) the treatment duration was short; (3) a low dose concentration or low bioavailability of pantethine. However, as pantethine supplementation can increase blood pantothenate concentrations, the combination of both pantothenate and pantethine can be more efficient in specific patients.

Signs of oxidative and increased ROS production after iron exposure have been previously reported in PKAN cellular models [72]. Consistent with these findings, Alvarez-Cordoba et al., found increased content of carbonylated proteins and mitochondrial lipid peroxidation in PKAN fibroblasts [19]. Lipid peroxidation is generally described as a chain reaction caused by the oxidative damage of polyunsaturated fatty acids (PUFA) resulting in the generation of lipid peroxyl radicals, hydroperoxides and aldehyde derivatives [31]. Three stages are described during the process of lipid peroxidation: initiation, propagation, and termination [152]. The chemical reactions associated with each of these steps can be found elsewhere [153]. Peroxidation of lipids can disturb the assembly of the membrane, causing alterations in fluidity, permeability and ion transport [154]. Furthermore, many breakdown metabolites, such as malondialdehyde (MDA) and 4-hydroxynonenal (4-HNE) are generated in this process [155]. MDA and 4-HNE protein and DNA adducts modify multiple cellular processes and participate in secondary crosslinking reactions which may worsen the pathophysiology of the disease. In addition, lipid aldehydes may affect protein kinases and phosphatase activities leading to the abnormal activity of various transcription factors involved in cellular homeostasis [156].

Lipid peroxidation in organelles with high iron content, such as mitochondria, and alteration in membrane-dependent cellular processes such as vesicle trafficking and/or autophagy/mitophagy, can cause iron accumulation in lipofuscin granules, which in turn increases lipid peroxidation of membranes [156]. This vicious cycle of events that augment each other may aggravate and precipitate the progression of neurodegenerative diseases such as PKAN. Membrane antioxidants, such as vitamin E, can block this vicious cycle in neurodegenerative diseases by stopping lipid peroxidation propagation [157].

In addition, vitamin E is a necessary nutrient for neural development and neurological function [158]. This fact, together with much evidence demonstrating that neurodegenerative diseases are associated with oxidative stress and lipid peroxidation, leads to the hypothesis that the progression of neurodegeneration may be mitigated by membrane antioxidants such as vitamin E [159]. Several works in human and animal models of vitamin E deficiency assessed its participation in protecting the brain, and more specifically the cerebellum, from oxidative damage [160].

Lipid peroxidation has been related to the initiation and progression of many neurodegenerative disorders, including Alzheimer’s disease (AD), Parkinson’s disease (PD), and amyotrophic lateral sclerosis (ALS) [161]. Likewise, PKAN’s pathomechanisms are directly related to the overproduction of ROS and mitochondrial redox imbalance [162]. Particularly, lipid peroxidation and increased ROS production have been detected in fibroblast and iNs derived from PKAN’s patients, [41,72]. Thus, the inhibition of lipid peroxidation propagation might slow the course and ameliorate the severity of PKAN disease.

The positive effects of omega-3 fatty acids treatment in many disorders are now well known by many studies assessing their implication in multiple biochemical functions, including the improvement of antioxidant defenses [163], the synthesis of anti-inflammatory factors, increased cellular membranes fluidity, and the modulation of gene expression [164,165,166]. Interestingly, it has been reported that omega-3 fatty acids supplementation also has antioxidant effects by suppressing lipid peroxidation [167]. In addition, they have been implicated in synaptic plasticity, contributing to the enhancement of cognitive activity [164]. Scientific evidence is accumulating on the potential efficacy of omega-3 fatty acids treatment in neurodegenerative diseases in general [168,169], and in AD and PD in particular [170].

α-Lipoic acid is a pleiotropic organosulfur compound necessary for mitochondrial activity and energy generation, as well as for regulating gene expression [171,172,173]. α-Lipoic acid is produced from plants, animals, and humans and is synthesized de novo in mitochondria using mtFAS II, S-adenosylmethionine, and iron-sulfur group intermediates [173]. α-Lipoic acid has a determinant role in oxidative metabolism characterized by its antioxidant properties; this is the reason why it has neuroprotective and anti-inflammatory properties [174]. In this respect, α-lipoic acid can decrease the levels of proinflammatory molecules and eliminate ROS and reactive nitrogen species (RNS) [175]. In addition, α-lipoic acid supplementation has been shown to reduce lipid peroxidation and increase cellular antioxidant activity [176].

From an energetic point of view, α-lipoic acid acts as a cofactor for pyruvate dehydrogenase (PDH), α-ketoglutarate dehydrogenase (KDH), protein H of the glycine cleavage system (GCS) and branched-chain ketoacid dehydrogenase [177,178,179]. Furthermore, several studies have demonstrated that α-lipoic acid also has chelating properties on metals such as iron or copper and a positive impact on oxidative stress and lipid peroxidation [180]. These findings suggest that α-lipoic acid is an interesting compound for the treatment of neurodegenerative diseases such as PKAN. Corroborating this hypothesis, α-lipoic acid supplementation decreased significantly iron accumulation in responsive PKAN fibroblasts and iNs [123]. These results are also consistent with the positive effect of α-lipoic acid supplementation on reducing the age-dependent iron overload in the rat cerebral cortex [181]. Moreover, α-lipoic acid also avoided iron overload caused by ferric ammonium citrate supplementation in a zebrafish model [182].

In summary, antioxidants such as vitamin E, omega 3 and α-lipoic acid can protect cell membranes from oxidative stress and lipid peroxidation, a principal pathological feature present in PKAN [19,32] and other NBIA disorders [117].

On the other hand, L-carnitine, a quaternary amine (3-hydroxy-4-N-trimethylaminobutyrate) that is synthesized from the amino acids lysine and methionine, is necessary for the translocation of fatty acids to the mitochondrial compartment for β-oxidation. In addition, L-carnitine has a role in carbohydrate metabolism, stimulates mitochondrial biogenesis by increasing gene expression of mitochondrial components, and prevents the accumulation of toxic products or reactive radicals [183,184]. Mitochondrial dysfunction in PKAN may impair fatty acid β-oxidation which can preferentially affect brain metabolism. Furthermore, dysfunction of the mitochondrial respiratory chain provokes an increase in the NADH/NAD (+) ratio that inhibits β-oxidation and secondarily L-carnitine deficiency [185]. Therefore, L-carnitine as a natural compound that can increase cellular energy production may have therapeutic potential in PKAN. Recently, many works have shown the positive effects of L-carnitine supplementation on mitochondrial function in several pathologies [184,186].

Furthermore, as PDH deficiency is a major pathologic feature of PKAN, PDH-enhancing agents such as thiamine [187] may act as an interesting adjuvant therapy. Thiamine has many functions in cell metabolism since it functions as a cofactor of several multimeric enzymes such as PDH and α-KGDH complexes that participate in the Krebs cycle. In addition, it has been described that thiamine treatment has positive effects in several patients with PDH deficiency due to pyruvate dehydrogenase alpha subunit (E1) mutations [188,189,190,191,192].

Interestingly, all positive compounds identified after personalized drug screens (pantothenate, pantethine, vitamin E, omega 3, α-lipoic acid, L-carnitine, and thiamine) increased PANK2 transcripts and protein expression levels and up-regulated key transcription factors such as NF-Y, FOXN4, and hnRNPA/B [122,123] which are involved in *PANK2* gene expression [193]. Furthermore, it is known that these positive supplements also activate mitochondrial biogenesis through the expression of essential regulators such as peroxisome proliferator-activated receptor coactivator-1α (PGC1α) and mitochondrial transcription factor A TFAM [194,195,196]. Taken together, these data provide useful information on the molecular mechanisms involved in the positive effect of pantothenate, pantethine, vitamin E, α-lipoic acid, omega 3, L-carnitine, and thiamine.

It is hypothesized that partial correction of PANK2 expression levels by these compounds may increase CoA biosynthesis in the mitochondrial compartment, allowing 4′-phosphopantethenylation of essential mitochondrial proteins such as mtACP, mitochondrial10-FTHFDH (ALDH1L2) and AASS [20]. In agreement with this hypothesis, the results showed that the expression levels of several 4′-phosphopantetheine carrier proteins in PKAN cells were increased in responsive pathogenic variants after pantothenate, pantethine, vitamin E, omega 3, α-lipoic acid, L-carnitine or thiamine supplementation [122,123].

## 5. Polytarget Therapy in PKAN

Since several compounds have a positive effect on PKAN cell models, an interesting approach would be to examine their therapeutic efficacy both individually or in combination in controlled clinical trials. In fact, the strategy of combining several compounds that simultaneously affect different cellular pathways or processes are standard procedure in many important therapeutic areas such as cancer, Alzheimer’s disease (AD), Parkinson’s disease (PD), inflammation, epilepsy, depression, and other psychiatric disorders and may be more effective in controlling complex diseases such as PKAN [197,198,199]. Disadvantages of monotherapies can thus be overcome by designing drug combinations that modulate multiple targets [200].

Cellular models derived from patients with genetic neurodegenerative diseases allow for the systematic identification of drugs and their potential synergistic combinations that can rapidly move into preclinical development and clinical practice [201,202].

The progression of neurodegenerative diseases contributes to various factors such as mitochondrial dysfunction, iron accumulation, oxidative stress, inflammation, as well as genetic and environmental factors [203]. Therefore, multitargeted therapies with antioxidant and mitochondrial-stimulating compounds may address the multifactorial and complex nature of these diseases more effectively [204,205]. Multitarget therapeutic approaches have recently become a useful strategy in the development of potential treatments for neurological disorders [206].

However, since the crossing of substances to the brain depends on transport mechanisms present in the blood-brain barrier and the diffusion of these compounds also depends on the physicochemical characteristics of the molecule, further studies are warranted on the clinical effects of the positive compounds considering its bioavailability, pharmacokinetics and, in particular, its transport through the blood-brain barrier [207].

## 6. Conclusions

Cellular models derived from PKAN patients are useful tools both for understanding the underlying pathological mechanisms of the disease and for carrying out polytarget pharmacological screenings that make it possible to identify compounds and their combinations capable of correcting the mutant phenotype.

Genomics, transcriptomics, proteomics, and metabolomics complemented by the analysis of the response of patient-derived cells to different treatments will provide key information for a more rational therapeutic approach in complex diseases such as PKAN. In this way, treatments for PKAN disease could be optimized considering the specific pathological variants and the response of patient-derived cells to available therapies.

## Data Availability

Data sharing is not applicable.
